# The relationship between alcohol use and long-term cognitive decline in middle and late life: a longitudinal analysis using UK Biobank

**DOI:** 10.1093/pubmed/fdx186

**Published:** 2018-01-09

**Authors:** Giovanni Piumatti, Simon C Moore, Damon M Berridge, Chinmoy Sarkar, John Gallacher

**Affiliations:** 1 Department of Psychiatry, University of Oxford, Oxford, UK; 2 Unit of Development and Research in Medical Education (UDREM), Faculty of Medicine, University of Geneva, Geneva, Switzerland; 3 Violence & Society Research Group, School of Dentistry, Cardiff University, Cardiff, UK; 4 Farr Institute—CIPHER, Swansea University Medical School, Swansea UK; 5 Healthy High Density Cities Lab, HKUrbanLab, The University of Hong Kong, Hong Kong, China

**Keywords:** alcohol, alcohol consumption, public health

## Abstract

**Background:**

Using UK Biobank data, this study sought to explain the causal relationship between alcohol intake and cognitive decline in middle and older aged populations.

**Methods:**

Data from 13 342 men and women, aged between 40 and 73 years were used in regression analysis that tested the functional relationship and impact of alcohol on cognitive performance. Performance was measured using mean reaction time (RT) and intra-individual variation (IIV) in RT, collected in response to a perceptual matching task. Covariates included body mass index, physical activity, tobacco use, socioeconomic status, education and baseline cognitive function.

**Results:**

A restricted cubic spline regression with three knots showed how the linear (*β*_1_ = −0.048, 95% CI: −0.105 to −0.030) and non-linear effects (*β*_2_ = 0.035, 95% CI: 0.007–0.059) of alcohol use on mean RT and IIV in RT (*β*_1_ = −0.055, 95% CI: −0.125 to −0.034; *β*_2_ = 0.034, 95% CI: 0.002–0.064) were significant adjusting for covariates. Cognitive function declined as alcohol use increased beyond 10 g/day. Decline was more apparent as age increased.

**Conclusions:**

The relationship between alcohol use and cognitive function is non-linear. Consuming more than one UK standard unit of alcohol per day is detrimental to cognitive performance and is more pronounced in older populations.

## Introduction

The neurodegenerative effects of excessive alcohol consumption are well documented.^1–4^ Alzheimer’s disease and dementia have replaced ischaemic heart disease as the leading cause of death in England and Wales,^5^ and death rates for neurological disease are increasing worldwide.^6–8^ A limited number of studies suggest a J- or U-shaped relationship between the volume of alcohol consumed and the long-term cognitive decline,^9–11^ suggesting light to moderate alcohol consumption is a positive predictor of health status in older adults,^12^ protects cognition and may reduce the risk of dementia^13–15^ in later life.

The suggested curvilinear association between alcohol and cognition, however, is controversial. Recent reviews^16–18^ and meta-analyses^19–21^ indicate that there is little consensus on the level of alcohol consumption at which the harmful effects of alcohol on cognition emerge. Furthermore, a Mendelian Randomization study of alcohol and cognitive performance found evidence of benefit from reduced alcohol intake at all levels of self-reported consumption.^22^

The current study examined the shape of the association between alcohol consumption and change in cognitive performance. Data were drawn from UK Biobank, a large cohort of middle and older aged adults. Respondents who consumed alcohol at least once a week or more frequently were eligible for inclusion to reduce selection and reporting biases. A reaction time (RT) task was used as a robust test of central processing speed. Cognitive performance was measured using mean RT and IIV in RT.

## Methods

### Sample

Between 2006 and 2010, a heterogeneous population sample of 502 649 adults aged 40–73 years participated in the UK Biobank prospective cohort study at 22 research centres located across the UK.^23^ Participants were registered with the UK National Health Service (NHS) and lived within a radius of 40 km from one of the research centres. Self-reported data were collected via touch screen questionnaires and interview.^23^ Information on the assessment procedure, protocol and information on data access is available online (www.ukbiobank.ac.uk). For the purposes of estimating regression dilution, 20 346 individuals underwent a repeat assessment five years after their initial assessment. Data from these respondents are used in the current longitudinal analysis. Individuals were omitted from the analysis if they disclosed a history of neurological disorder at either baseline or follow-up (Table S1), leaving 19 124 eligible participants. The UK Biobank study was approved by the North West Multi-Centre Research Ethics Committee (reference number 06/ MRE08/65). All participants gave written, informed consent.

### Measures

#### Alcohol use

Alcohol consumption was measured using the question ‘about how often do you drink alcohol?’ Available responses were ‘daily or almost daily’, ‘three of four times a week’, ‘once or twice a week’, ‘one to three times a month’, ‘special occasions only’, ‘never’ and ‘prefer not to answer’. Respondents who drank alcohol once a week or more frequently were asked to record how many alcoholic drinks they consumed on average each week from a list of common alcoholic beverages (red and white wine, champagne, beer and cider, spirits and liquors, fortified wine, and other alcoholic drinks), or to respond ‘do not know’ or ‘prefer not to answer’. Volumes were specified when referring to beverages (e.g. ‘there are six glasses in an average bottle of wine’; ‘there are 25 standard measures in a normal sized bottle’). Respondents who declared that they drank alcohol ‘one to three times a month’ or on ‘special occasions only’ (henceforth monthly drinkers) were also asked to record how many drinks they consumed on average each month. However, these questions were not included at baseline and therefore more than half of the sample of monthly drinkers at baseline were not assessed. Accordingly, only participants who declared that they drank at least once a week (henceforth, weekly drinkers) were included in primary analyses.

### Cognitive performance

Cognitive performance was assessed using a ‘stop-go’ RT task in which participants were shown two cards simultaneously on a computer screen. Each card had a symbol on it and participants were asked to respond as quickly as possible, using a button-box, if both symbols matched. RT, from the presentation of the cards to their press of the button, was recorded in milliseconds (ms). Each participant was presented with 12 pairs of cards, the first five of which were training sets and data from these trials were discarded. Of the seven test trials, cards with matching symbols were presented on four occasions selected at random. A demonstration of this test is available online (biobank.ctsu.ox.ac.uk/crystal/videos/snap.swf).

### Covariates

The effects of alcohol use on cognitive performance differ by gender,^24, 25^ education,^26^ past performance^26^ and age.^20^ These variables were included as covariates in the present study alongside deprivation as measured by the Townsend score,^27^ physical activity assessed as walking activity, body mass index (BMI) and smoking status.

### Data analysis

Alcohol consumption in grams per day was calculated by multiplying the average number of alcoholic drinks consumed each week by the average grams of alcohol contained in each type of drink, determined using the UK Food Standard Agency’s guidelines.^28^ The total was then divided by seven to provide mean daily alcohol consumption. Alcohol consumption was positively skew and log transformed.

Consistent with established methods,^29^ RTs < 50 ms, indicating anticipation of the stimulus, were discarded as were RTs > 2 s, as the target stimulus had been withdrawn at this point. RT was calculated as the arithmetic mean of completed test trials. Intra-individual variation (IIV) was calculated as the standard deviation of each participant’s RTs over the test trials.^30^ Participants with only one valid score at baseline or follow-up were omitted. RT and IIV showed log–normal distributions and were natural log transformed.

For the covariates, educational attainment was included as a binary variable (with or without a degree). BMI was included as two binary variables (normal <24.9 kg/m^2^ compared to overweight 25–29.9 kg/m^2^ and obese ≥30 kg/m^2^) as was smoking status (non-smoker compared to previous smoker and current smoker). Deprivation quintiles were included as a continuous variable, as were age and the time between baseline and follow-up assessments. Walking activity was included as the number of days participants walked for more than 10 min each week. Preliminary analyses found missing data was minimal (2.8%).

Non-linearity in the alcohol–cognition relationship was investigated using restricted cubic splines. A restricted cubic spline is a cubic spline function with an additional constraint of linearity before the first knot and after the last knot.^31^ The number of knots was determined by examining the distribution of average daily alcohol use in the sample, with the aim of locating boundaries between equal-sized categories and by comparing the Akaike Information Criteria (AIC) goodness of fit statistics across models.

Staged multivariable modelling, the regression of follow-up RT and IIV on baseline alcohol consumption, began with an adjustment for age to establish the fundamental association (model 1). Adjustment for social confounding was made by further conditioning on lifestyle and background (model 2). The influence of baseline cognition was then taken into account (model 3). Finally, interactions effects were included (model 4). All analyses were performed using Stata 14 (StataCorp. 2015. Stata Statistical Software: Release 14. College Station, TX: StataCorp LP).

## Results

Of the 19 124 individuals with follow-up data and no history of neurological disorder, there were 14 349 weekly drinkers. Of these, complete data were available for 13 342 (93%).

Weekly drinkers had lower levels of socioeconomic deprivation, were more likely to be male and to hold an undergraduate degree or higher. Non-drinkers were older and reported worse cognitive scores across time (Table S2). RT varied by age, gender, education, BMI, walking activity, alcohol consumption and smoking status (Table 1). IIV varied by age, gender, education, BMI, walking activity and alcohol consumption (Table 1).

**Table 1:** Differences in mean reaction time (RT) and intra-individual variation in reaction time (IIV) at follow-up according to socioeconomic and lifestyle factors. Values are means and (standard deviations)

Variable	RT (ms)	IIV
Age (years)		
40–52	512.76 (95.25)***	68.53 (49.38)***
53–59	551.78 (102.18)	77.15 (55.23)
60–63	571.90 (110.04)	82.27 (58.56)
64+	594.67 (114.15)	88.95 (66.03)
Gender		
Females	562.51 (109.01)***	79.80 (59.02)
Males	548.80 (108.74)	77.40 (55.70)
Education		
No degree	564.44 (112.48)***	81.25 (59.22)***
Degree	544.74 (103.67)	75.34 (54.98)
Deprivation quintile		
Least	553.77 (102.27)***	78.54 (56.85)***
2	555.94 (109.68)	78.69 (57.23)
3	555.49 (109.33)	79.72 (59.46)
4	558.15 (109.76)	78.54 (57.59)
Most	558.80 (113.94)	77.65 (56.12)
Alcohol intake		
Non-drinkers	574.79 (119.88)**	83.99 (67.08)**
Monthly	558.45 (112.07)	78.18 (56.34)
Weekly	553.84 (107.35)	78.38 (56.98)
Body mass index		
≤Normal	555.57 (109.10)***	78.72 (58.20)**
Overweight	553.86 (108.11)	77.45 (55.24)
Obese	561.73 (111.56)	81.26 (60.55)
Walking tertiles (days/week)		
0–4	550.89 (106.82)*	76.87 (54.65)**
5–6	555.28 (108.57)	78.35 (56.83)
7	559.50 (110.27)	80.02 (59.64)
Smoking		
Non-smokers	552.91 (108.06)***	78.22 (57.40)
Previous smokers	561.07 (110.88)	79.48 (57.78)
Current smokers	554.39 (107.61)	77.65 (55.23)

***P < 0.001, **P < 0.01, *P < 0.05.

### Curvilinear modelling

Preliminary analyses aimed to identify the most parsimonious curvilinear model. Models with knots at the 25th, 50th and 75th percentiles in the alcohol distribution provided the best fit for RT (AIC = −13 691, *F*(12, 13 329) = 475.54, *P* < 0.001) and IIV (AIC = 24,985, *F*(12, 13 329) = 53.28, *P* < 0.001). This curvilinear solution was superior to a linear model (RT: AIC = −13 687.48; IIV: AIC = 24 987.20), and models with two (RT: AIC = −13 687.48; IIV: AIC = 24 987.20) and four knots (RT: AIC = −13 690; IIV: AIC = 24 986.14).

**Fig. 1:**
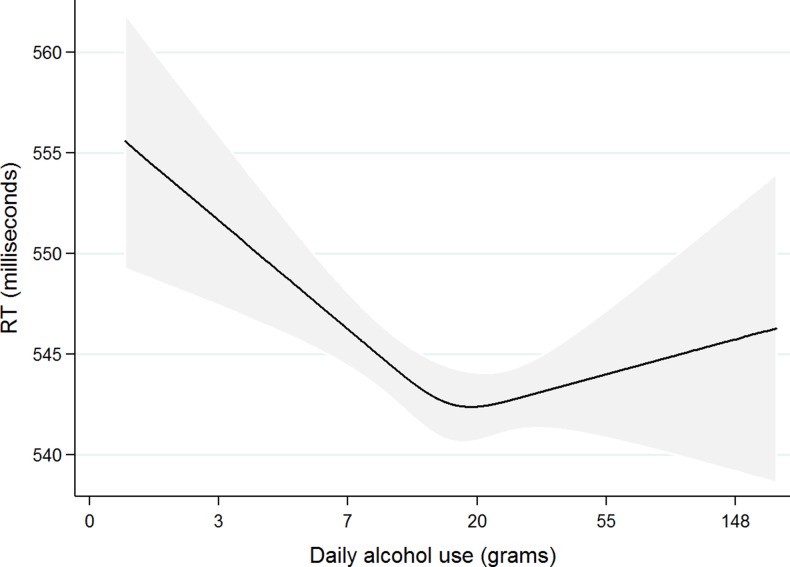
Curvilinear association between average daily alcohol use at baseline and mean reaction time (RT) at follow-up for the full sample, with 99% confidence intervals (*N* = 13 342). Estimates are adjusted for age, years between assessments, gender, education, Townsend deprivation score, smoking status, BMI, walking activity and RT at baseline.

RT was associated with baseline RT, alcohol consumption, age and years between assessments, gender, education and smoking status (Table 2). IIV was associated with baseline IIV, alcohol consumption, age, years between assessments and education (Table 2). RT decreased by 0.102 SD units (0.048 ms) for every additional 1 g/day increase in alcohol consumption up to 10 g/day, meaning cognitive performance improved. Cognitive performance declined as alcohol consumption increased beyond 10 g/day (Fig. 1). The limitations of the cubic spline method make it difficult to quantify the potential for harm, not least due to the relatively small numbers of heavy drinkers in the UK Biobank sample.

**Table 2:** Restricted cubic spline regression model results with baseline measures predicting mean reaction time (RT) and intra-individual variation in reaction time (IIV) at follow-up (*N* = 13 342)

Predictors	Outcomes *β* (95% CI) *P* value	
	RT	IIV
Mean reaction time baseline	0.548 (0.529 to 0.561) <0.001	–
Intra-individual variation at baseline	–	0.178 (0.161 to 0.196) <0.001
Alcohol use: spline 1 (linear effect ≤ 10 g/day)	−0.048 (−0.105 to −0.030) 0.001	−0.055 (−0.125 to −0.034) 0.001
Alcohol use: spline 2 (slope effect)	0.035 (0.007 to 0.059) 0.013	0.034 (0.002 to 0.064) 0.039
Age in years (at repeat assessment)	0.135 (0.072 to 0.107) <0.001	0.085 (0.072 to 0.107) <0.001
Gender (reference: female)	−0.023 (−0.037 to −0.008) 0.002	−0.005 (−0.023 to 0.013) 0.558
Education (reference: no degree)	−0.027 (−0.047 to −0.014) <0.001	−0.031 (−0.047 to −0.014) <0.001
Previous tobacco use (reference: non-smoker)	0.020 (0.005 to 0.033) 0.008	0.008 (−0.009 to 0.025) 0.336
Fit	*R*^2^ = 0.35; AIC = −13 691	*R*^2^ = 0.05; AIC = 24 985

B, unstandardized regression coefficient; SE B, standard error for the unstandardized regression coefficient; *β* (95% CI), standardized regression coefficient and 95% confidence intervals. Note: results for smoking, walking, BMI and deprivation omitted as did not approach statistical significance for either outcome

IIV decreased by −0.055 units for every additional 1 g/day increase in alcohol consumption up to 10 g/day, also indicating better performance at low alcohol levels but not at higher consumption levels. Multivariable modelling made no material difference to these associations (Table 3). Although adjustment for social covariates and baseline cognition marginally attenuated the association, statistical significance was retained.

**Table 3:** Restricted cubic regression of cognitive performance on daily alcohol consumption

Cognition	Alcohol consumption splines	Model 1: Adjusted for age *β* (95% CI) *P*-value	Model 2: Adjusted for age + covariates *β* (95% CI) *P*-value	Model 3: Adjusted for age + covariates + baseline cognition *β* (95% CI) *P*-value
RT	Linear (Spline 1: Linear effect up to 10 g/day)	−0.102 (−0.187 to −0.099)	−0.098 (−0.183 to −0.093)	−0.048 (−0.105 to −0.030)
		<0.001	<0.001	<0.001
	Non-linear (Spline 2: Slope effect)	0.056 (0.023–0.083)	0.055 (0.021–0.083)	0.035 (0.007–0.059)
		0.001	0.001	0.013
IIV	Linear (Spline 1: Linear effect up to 10 g/day)	−0.064 (−0.137 to −0.046)	−0.064 (−0.138 to −0.045)	−0.055 (−0.125 to −0.034)
		<0.001	<0.001	0.001
	Non-linear (Spline 2: Slope effect)	0.042 (0.009–0.072)	0.038 (0.005–0.068)	0.034 (0.002–0.064)
		0.011	0.024	0.039

RT, mean reaction time; IIV, intra-individual variability in reaction time; *β* (95% CI), standardized regression coefficient and 95% confidence intervals.

The model was refitted with interaction terms for age, gender, education, deprivation, smoking status, BMI and baseline cognition. For RT, age made little difference to the linear effect, i.e. potential benefit incurred below 10 g/day (*β*_1_ = 0.104, 95% CI: 0.101, 0.176) but moderately increased the non-linear effect, i.e. potential harm incurred above 10 g/day (*β*_2_ = −0.070, 95% CI: −0.093, −0.039) (Table S4). A similar effect was found for IIV.

## Discussion

### Main finding of this study

In 13 342 weekly drinkers drawn from UK Biobank, 5-year change in mean RT and IIV in RT were found to have curvilinear associations with alcohol consumption. Cognitive performance improved as alcohol consumption increased up to 10 g/day and then deteriorated as alcohol consumption increased beyond 10 g/day. As individuals age, this deleterious effect of alcohol on cognitive performance became more pronounced.

### What is already known on this topic

The long-term impact of alcohol use on cognition is controversial. Observational epidemiologic data of alcohol consumption and the incidence of cognitive impairment and dementia show reduced risk with light to moderate alcohol consumption.^19, 32, 33^ Studies of alcohol consumption and cognitive decline have reported a reduced rate of decline in light and moderate drinkers compared to abstainers and heavy drinkers.^11, 34^

However, evidence against a ‘J’ shaped relationship accumulates. A Mendelian randomization instrumental variable analysis in a Chinese population^22^ compared alcohol consumption according to ADHD2 variants known to be associated with alcohol consumption. A per-allele association with cognitive performance between ADHD2 variants was not found. Although this study was underpowered, and heavy drinkers (by Western standards) were absent from the sample, the finding is consistent with a large-scale Mendelian randomization study in a Western population. Holmes *et al.*^35^ failed to find evidence for a ‘J’ shaped association between alcohol and cardiovascular risk, a condition that shares many if the mechanisms underlying cerebrovascular risk.

### What this study adds

This study presents data on cognitive change at an individual level across a wide range of alcohol consumption, in contrast to data on cognitive differences between alcohol consumption groups. It also omits abstainers. Both design features ameliorate the impact of reverse causation on the findings. The use of a RT task, conducted in a standard and controlled environment, provided a precise and reliable measure of cognitive performance. Treating alcohol consumption as a continuous variable facilitated a dose–response analysis. These findings do not resolve the debate over whether benefit may be attributed to low level alcohol consumption. If there is no benefit, these findings demonstrate that adjusting adequately for confounding on this question is extremely difficult. If there is benefit, the mechanisms remain obscure.

Given uncertainties concerning the shape of the association there is a strong case for changing the focus of the debate to harm rather than benefit. There is little question that alcohol is neurotoxic and that no cognitive benefit derives from high consumption levels. The findings reported here indicate that harm becomes apparent at levels of alcohol consumption lower than previously reported. Zuccalà *et al.*,^36^ for example, argue for a protective effect of wine up to 40 g/day for women and up to 80 g/day for men. Britton *et al.*^37^ suggest that the beneficial effects of alcohol among UK middle-aged adults occur up to 34 g/day, whilst UK department of Health guidelines are that drinkers should not consume more than 16 g/day to minimize the risk of alcohol to health. Our findings suggest that to preserve cognitive performance 10 g/day is a more appropriate upper limit. This would translate into not more than one UK standard unit of alcohol each day. Our findings are of particular relevance to older individuals who demonstrated a greater rate of decline as alcohol consumption increased.

### Limitations of the study

Statistical limitations require consideration. The restricted spline method enables the inflexion point in the curve to be identified but assumes linearity before and after the inflexion. This assumption is unlikely to make much impact below the inflexion point due to the limited scale range (the proximity of zero), but it is a strong assumption above the inflexion point. The wide confidence intervals on the curve above the inflexion (Fig. 1) indicate that further work is required to reduce uncertainty in the functional relationship between cognitive performance and alcohol consumption above 10 g/day.

The ‘J’ shaped association reported here should be considered critically. To reduce the ‘sick quitter’ effect abstainers were omitted. However, participants who may have only reduced alcohol intake for health reasons rather than quit, remain in the analysis. Selection bias may also be operating at high levels of alcohol consumption in that ‘bright boozers’, those with high alcohol intake and high cognitive performance, may be over represented at recruitment and follow-up, thus deflating estimates of harm at high levels of consumption. The extent to which this effect is ameliorated by heavy drinkers disproportionately under reporting consumption levels is also unknown. The effect of these selection and reporting biases is likely to be complex, but unlikely to materially affect the conclusion that alcohol consumption deleteriously affects cognitive performance at lower intake levels than previously thought.

### Further work

The extent to which the association of alcohol with cognition reported here may be generalized to other cognitive domains is interesting. Due to its precision of measurement, RT is likely to be the most sensitive of the cognitive measures used in UK Biobank. Higher cognitive domains, such as reasoning or memory, may provide greater opportunity for compensatory mechanisms to mask neurological impacts of alcohol consumption, particularly at low intakes. Nevertheless, the literature suggests the effect of alcohol on cognition is broad.^19, 38, 39^

The issue of alcohol consumption pattern is not addressed in these data and single session heavy episodic alcohol consumption may deliver an additional cognitive burden. Future studies are needed to test the differential or joint role of average volume versus drinking pattern in order to better understand the nature of the relationship between alcohol use and cognitive decline. A core methodological limitation is the use of self-report measures of alcohol consumption. Objective measures of alcohol consumption, such as metabolomic markers, are required to improve the rigour of alcohol intake assessment.^40^

## Conclusions

Current advice from the UK Department of Health^41^ is for men and women to not consume more than 16 g of pure alcohol per day (two units) on average. Findings reported here suggest that daily alcohol consumption above one unit is may have an adverse cognitive impact. Recommendations should be sensitive to this, especially among middle-aged and older members of the population.

## Supplementary Material

Supplementary Data

## Data sharing

This research has been conducted using the UK Biobank Resource under Application Number 14 935. The data reported in this article are available via application directly to the UK Biobank.
