# Supplementary material for: The relationship between alcohol use and long-term cognitive decline in middle and late life: a longitudinal analysis using UK Biobank
Source: J Public Health (Oxf). 2018 Jan 9;40(2):304–11. doi: 10.1093/pubmed/fdx186 (PMC6051452; doi:10.1093/pubmed/fdx186)
Supplement: Supplementary Data [file fdx186supplemental_materials.docx]

**Supplemental Materials**

**Contents**

**Table S1. Excluded self-reported neurological diseases.**

**Table S2. Baseline sociodemographic characteristics, longitudinal alcohol use and cognitive scores of 19,124 participants by frequency of alcohol consumption. Values are numbers (percentages) unless stated otherwise.**

**Table S3. Linear multiple regression model results with baseline measures predicting mean reaction time (RT) at follow-up (N=13,342). Categorical variables are reported with the reference category (RC) in brackets.**

**Table S4. Linear multiple regression model results with baseline measures predicting intra-individual variation in reaction time (IIV) at follow-up (N=13,342). Categorical variables are reported with the reference category (RC) in brackets.**

**Table S5. Restricted cubic spline regression model results and test of interaction effects with baseline measures predicting mean reaction time (RT) at follow-up (N=13,342). Categorical variables are reported with the reference category (RC) in brackets.**

**Table S6. Restricted cubic spline regression model results and test of interaction effects with baseline measures predicting intra-individual variation in reaction time (IIV) at follow-up (N=13,342). Categorical variables are reported with the reference category (RC) in brackets.**

**Table S1.**

**Excluded self-reported neurological diseases.**

| Brain cancer/primary malignant tumour  Brain haemorrhage  Brain/intracranial abscess  Cerebral aneurysm  Cerebral palsy  Chronic/degenerative neurological problem  Dementia/Alzheimer’s disease/cognitive impairment  Encephalitis  Epilepsy  Head injury  Infection of nervous system  Ischaemic stroke  Meningeal cancer/malignant meningioma  Meningioma (benign)  Meningitis  Motor neurone disease  Multiple sclerosis  Neurological injury/trauma  Neuroma (benign)  Other demyelinating condition  Other neurological problem  Parkinson’s disease  Spina bifida  Stroke  Subarachnoid haemorrhage  Subdural haematoma  Transient ischaemic attack |
| --- |

**Table S2.**

**Baseline sociodemographic characteristics, longitudinal alcohol use and cognitive scores of 19,124 participants by frequency of alcohol consumption. Values are numbers (percentages) unless stated otherwise.**

| **Variables** | **Weekly**  **drinkers**  **(n=14,349)** | **Monthly**  **drinkers**  **(n=3,724)** | **Previous**  **drinkers**  **(n=468)** | **Non**  **drinkers**  **(n=575)** | **P value** |
| --- | --- | --- | --- | --- | --- |
| **Age *Mean* (*SD*)** | 57.64 (7.34) | 56.94 (7.65) | 57.23 (7.57) | 57.89 (7.81) | <0.001 (F=9.54) |
| **Women** | 6,761 (47) | 2,466 (66) | 261 (56) | 386 (67) | <0.001 (χ^2^=492.87) |
| **Undergraduate degree or higher** | 6,445 (46) | 1,349 (37) | 170 (38) | 220 (39) | <0.001 (χ^2^=108.63) |
| **Smoking status:** |  |  |  |  | <0.001 (χ^2^=416.78) |
| Non smokers | 8,046 (56) | 2,545 (68) | 237 (51) | 508 (89) |  |
| Previous smokers | 5,374 (38) | 953 (26) | 196 (42) | 50 (9) |  |
| Smokers | 895 (6) | 221 (6) | 33 (7) | 16 (3) |  |
| **BMI categories:** |  |  |  |  | <0.001 (χ^2^=140.68) |
| Normal weight | 5315 (38) | 1,283 (35) | 168 (36) | 228 (40) |  |
| Overweight | 6,231 (44) | 1,421 (39) | 177 (38) | 199 (35) |  |
| Obese | 2,610 (18) | 969 (26) | 121 (26) | 141 (25) |  |
| **Townsend deprivation score *Mean* (*SD*)** | -2.19 (2.57) | -1.70 (2.90) | -1.36 (3.08) | -1.28 (3.11) | <0.001 (F=60.23) |
| **Walking activity (days/week) *Mean* (*SD*)** | 5.22 (1.98) | 5.12 (2.07) | 5.10 (2.17) | 5.34 (1.97) | 0.006 (F=4.21) |
| **Daily alcohol use (grams) at baseline *Mean* (*SD*)** | 21.77 (19.65) | - | - | - | - |
| **Daily alcohol use (grams) at follow-up *Mean* (*SD*)** | 18.80 (17.03) | - | - | - | - |
| **Years between assessments *Mean* (*SD*)** | 4.31 (0.92) | 4.36 (0.92) | 4.39 (0.95) | 4.40 (0.93) | <0.001 (F=5.63) |
| **RT at baseline *Mean* (*SD*)** | 546.15 (101.35) | 551.62 (103.70) | 562.56 (106.58) | 576.31 (127.12) | <0.001 (F=20.34) |
| **RT at follow-up *Mean* (*SD*)** | 553.84 (107.35) | 558.45 (112.07) | 565.58 (108.93) | 582.30 (127.72) | <0.001 (F=14.74) |
| **IIV at baseline *Mean* (*SD*)** | 76.55 (54.84) | 77.93 (56.30) | 79.68 (61.72) | 90.01 (81.64) | <0.001 (F=10.79) |
| **IIV at follow-up *Mean* (*SD*)** | 78.37 (56.96) | 78.18 (56.34) | 80.86 (61.58) | 86.54 (71.21) | <0.01 (F=3.99) |

RT=Mean reaction time; IIV=Intra-individual variability in reaction time.

**Table S3.**

**Linear multiple regression model results with baseline measures predicting mean reaction time (RT) at follow-up (N=13,342). Categorical variables are reported with the reference category (RC) in brackets.**

| **Predictors** | **RT** | | | |
| --- | --- | --- | --- | --- |
|  | **B** | **SE B** | **β (95% CI)** | **P value** |
| **RT at baseline** | 0.570 | 0.009 | 0.549 (0.530 to 0.562) | <0.001 |
| **Daily alcohol use** | -0.004 | 0.002 | -0.019 (-0.049 to -0.005) | 0.015 |
| **Age with years between assessments** | 0.001 | 0.001 | 0.135 (0.125 to 0.154) | <0.001 |
| **Male (RC: Female)** | -0.008 | 0.003 | -0.021 (-0.035 to -0.007) | 0.004 |
| **Townsend deprivation score** | -0.001 | 0.001 | -0.007 (-0.022 to 0.007) | 0.330 |
| **Academic degree or higher (RC: No academic degree)** | -0.010 | 0.003 | -0.028 (-0.040 to -0.013) | <0.001 |
| **Walking activity** | 0.001 | 0.001 | 0.011 (-0.002 to 0.024) | 0.103 |
| **Overweight BMI (RC: Normal or underweight)** | -0.004 | 0.003 | -0.010 (-0.024 to 0.005) | 0.211 |
| **Obese BMI (RC: Normal or underweight)** | 0.001 | 0.004 | 0.002 (-0.013 to 0.019) | 0.713 |
| **Previously used tobacco (RC: Non-smoker)** | 0.007 | 0.003 | 0.020 (0.005 to 0.033) | 0.006 |
| **Uses tobacco (RC: Non-smoker)** | 0.005 | 0.006 | 0.007 (-0.017 to 0.018) | 0.354 |
| **Fit** | R^2^=0.35; AIC=-13,687.48 | | | |

B=Unstandardized regression coefficient; SE B=Standard error for the unstandardized regression coefficient; β (95% CI)=Standardized regression coefficient and 95% Confidence Intervals.

**Table S4.**

**Linear multiple regression model results with baseline measures predicting intra-individual variability in reaction time (IIV) at follow-up (N=13,342). Categorical variables are reported with the reference category (RC) in brackets.**

| **Predictors** | **IIV** | | | |
| --- | --- | --- | --- | --- |
|  | **B** | **SE B** | **β (95% CI)** | **P value** |
| **IIV at baseline** | 0.177 | 0.009 | 0.178 (0.161 to 0.196) | <0.001 |
| **Daily alcohol use** | -0.022 | 0.007 | -0.027 (-0.065 to -0.013) | 0.003 |
| **Age with years between assessments** | 0.001 | 0.001 | 0.085 (0.072 to 0.108) | <0.001 |
| **Male (RC: Female)** | -0.005 | 0.012 | -0.004 (-0.022 to 0.014) | 0.663 |
| **Townsend deprivation score** | 0.001 | 0.002 | 0.001 (-0.016 to 0.019) | 0.907 |
| **Academic degree or higher (RC: No academic degree)** | -0.040 | 0.011 | -0.031 (-0.048 to -0.015) | <0.001 |
| **Walking activity** | 0.004 | 0.003 | 0.013 (-0.003 to 0.030) | 0.115 |
| **Overweight BMI (RC: Normal or underweight)** | -0.010 | 0.012 | -0.008 (-0.026 to 0.011) | 0.426 |
| **Obese BMI (RC: Normal or underweight)** | 0.018 | 0.016 | 0.011 (-0.008 to 0.031) | 0.252 |
| **Previously used tobacco (RC: Non-smoker)** | 0.011 | 0.012 | 0.009 (-0.009 to 0.026) | 0.340 |
| **Uses tobacco (RC: Non-smoker)** | 0.001 | 0.023 | 0.001 (-0.017 to 0.018) | 0.951 |
| **Fit** | R^2^=0.05; AIC=24,987.20 | | | |

B=Unstandardized regression coefficient; SE B=Standard error for the unstandardized regression coefficient; β (95% CI)=Standardized regression coefficient and 95% Confidence Intervals.

**Table S5.**

**Restricted cubic spline regression model results and test of interaction effects with baseline measures predicting mean reaction time (RT) at follow-up (N=13,342). Categorical variables are reported with the reference category (RC) in brackets.**

| **Predictors** | **RT** | | | |
| --- | --- | --- | --- | --- |
|  | **B** | **SE B** | **β (95% CI)** | **P value** |
| **RT at baseline** | 0.566 | 0.010 | 0.547 (0.523 to 0.562) | <0.001 |
| **Daily alcohol use (Spline 1: Linear effect up to 10g/day)** | -0.012 | 0.004 | -0.051 (-0.116 to -0.028) | 0.001 |
| **Daily alcohol use (Spline 2: Slope effect)** | 0.009 | 0.005 | 0.032 (-0.002 to 0.062) | 0.069 |
| **Age with years between assessments** | 0.001 | 0.001 | 0.120 (0.108 to 0.140) | <0.001 |
| **Male (RC: Female)** | -0.009 | 0.004 | -0.026 (-0.044 to -0.006) | 0.009 |
| **Townsend deprivation score** | -0.001 | 0.001 | -0.013 (-0.032 to 0.005) | 0.163 |
| **Academic degree or higher (RC: No academic degree)** | -0.010 | 0.003 | -0.028 (-0.044 to -0.010) | 0.002 |
| **Walking activity** | 0.001 | 0.001 | 0.001 (-0.015 to 0.016) | 0.909 |
| **Overweight BMI (RC: Normal or underweight)** | -0.002 | 0.003 | -0.006 (-0.022 to 0.010) | 0.463 |
| **Obese BMI (RC: Normal or underweight)** | 0.003 | 0.004 | 0.007 (-0.012 to 0.026) | 0.462 |
| **Previously used tobacco (RC: Non-smoker)** | 0.006 | 0.003 | 0.016 (-0.002 to 0.033) | 0.091 |
| **Uses tobacco (RC: Non-smoker)** | 0.005 | 0.007 | 0.007 (-0.013 to 0.026) | 0.516 |
| **Daily alcohol use Spline 1 with RT at baseline** | -0.006 | 0.005 | -0.023 (-0.081 to 0.018) | 0.214 |
| **Daily alcohol use Spline 2 with RT at baseline** | 0.007 | 0.003 | 0.040 (0.005 to 0.070) | 0.022 |
| **Daily alcohol use Spline 1 with age** | 0.026 | 0.004 | 0.104 (0.101 to 0.176) | <0.001 |
| **Daily alcohol use Spline 2 with age** | -0.012 | 0.003 | -0.07 (-0.093 to -0.039) | <0.001 |
| **Daily alcohol use Spline 1 with gender** | 0.001 | 0.004 | 0.002 (-0.038 to 0.043) | 0.887 |
| **Daily alcohol use Spline 2 with gender** | 0.001 | 0.003 | 0.008 (-0.021 to 0.037) | 0.609 |
| **Daily alcohol use Spline 1 with Townsend deprivation score** | 0.003 | 0.004 | 0.013 (-0.029 to 0.064) | 0.453 |
| **Daily alcohol use Spline 2 with Townsend deprivation score** | -0.001 | 0.003 | -0.003 (-0.034 to 0.029) | 0.873 |
| **Daily alcohol use Spline 1 with education** | -0.001 | 0.004 | -0.001 (-0.042 to 0.040) | 0.969 |
| **Daily alcohol use Spline 2 with education** | 0.002 | 0.003 | 0.001 (-0.018 to 0.037) | 0.505 |
| **Daily alcohol use Spline 1 with walking activity** | -0.002 | 0.003 | -0.006 (-0.043 to 0.026) | 0.639 |
| **Daily alcohol use Spline 2 with walking activity** | 0.003 | 0.002 | 0.016 (-0.009 to 0.040) | 0.223 |
| **Daily alcohol use Spline 1 with BMI** | -0.003 | 0.004 | -0.010 (-0.059 to 0.028) | 0.479 |
| **Daily alcohol use Spline 2 with BMI** | 0.003 | 0.003 | 0.015 (-0.013 to 0.041) | 0.312 |
| **Daily alcohol use Spline 1 with tobacco use** | -0.001 | 0.004 | -0.003 (-0.051 to 0.043) | 0.87 |
| **Daily alcohol use Spline 2 with tobacco use** | 0.001 | 0.005 | 0.007 (-0.026 to 0.038) | 0.705 |
| **Fit** | R^2^=0.35; AIC=-12542.42 | | | |

B=Unstandardized regression coefficient; SE B=Standard error for the unstandardized regression coefficient; β (95% CI)=Standardized regression coefficient and 95% Confidence Intervals.

**Table S6.**

**Restricted cubic spline regression model results and test of interaction effects with baseline measures predicting intra-individual variability in reaction time (IIV) at follow-up (N=13,342). Categorical variables are reported with the reference category (RC) in brackets.**

| **Predictors** | **IIV** | | | |
| --- | --- | --- | --- | --- |
|  | **B** | **SE B** | **β (95% CI)** | **P value** |
| **IIV at baseline** | 0.178 | 0.010 | 0.179 (0.160 to 0.200) | <0.001 |
| **Daily alcohol use (Spline 1: Linear effect up to 10g/day)** | -0.039 | 0.014 | -0.050 (-0.121 to -0.022) | 0.005 |
| **Daily alcohol use (Spline 2: Slope effect)** | 0.020 | 0.019 | 0.020 (-0.018 to 0.057) | 0.310 |
| **Age with years between assessments** | 0.001 | 0.001 | 0.076 (0.061 to 0.098) | <0.001 |
| **Male (RC: Female)** | -0.014 | 0.014 | -0.011 (-0.033 to 0.011) | 0.341 |
| **Townsend deprivation score** | -0.001 | 0.002 | -0.006 (-0.026 to 0.015) | 0.579 |
| **Academic degree or higher (RC: No academic degree)** | -0.045 | 0.013 | -0.035 (-0.055 to -0.015) | 0.001 |
| **Walking activity** | 0.005 | 0.003 | 0.015 (-0.004 to 0.035) | 0.117 |
| **Overweight BMI (RC: Normal or underweight)** | -0.008 | 0.013 | -0.006 (-0.025 to 0.013) | 0.549 |
| **Obese BMI (RC: Normal or underweight)** | 0.018 | 0.017 | 0.011 (-0.010 to 0.034) | 0.299 |
| **Previously used tobacco (RC: Non-smoker)** | 0.011 | 0.014 | 0.008 (-0.012 to 0.028) | 0.437 |
| **Uses tobacco (RC: Non-smoker)** | 0.005 | 0.029 | 0.002 (-0.020 to 0.024) | 0.858 |
| **Daily alcohol use Spline 1 with IIV at baseline** | -0.012 | 0.015 | -0.015 (-0.067 to 0.027) | 0.411 |
| **Daily alcohol use Spline 2 with IIV at baseline** | 0.005 | 0.010 | 0.008 (-0.024 to 0.040) | 0.624 |
| **Daily alcohol use Spline 1 with age** | 0.058 | 0.014 | 0.067 (0.050 to 0.135) | <0.001 |
| **Daily alcohol use Spline 2 with age** | -0.022 | 0.010 | -0.034 (-0.063 to -0.004) | 0.026 |
| **Daily alcohol use Spline 1 with gender** | 0.011 | 0.013 | 0.015 (-0.029 to 0.063) | 0.461 |
| **Daily alcohol use Spline 2 with gender** | -0.002 | 0.012 | -0.003 (-0.037 to 0.032) | 0.887 |
| **Daily alcohol use Spline 1 with Townsend deprivation score** | 0.024 | 0.015 | 0.028 (-0.011 to 0.090) | 0.126 |
| **Daily alcohol use Spline 2 with Townsend deprivation score** | -0.009 | 0.010 | -0.015 (-0.047 to 0.017) | 0.362 |
| **Daily alcohol use Spline 1 with education** | 0.009 | 0.015 | 0.010 (-0.033 to 0.060) | 0.564 |
| **Daily alcohol use Spline 2 with education** | 0.002 | 0.010 | 0.004 (-0.028 to 0.035) | 0.811 |
| **Daily alcohol use Spline 1 with walking activity** | -0.012 | 0.015 | -0.015 (-0.066 to 0.027) | 0.405 |
| **Daily alcohol use Spline 2 with walking activity** | 0.011 | 0.010 | 0.018 (-0.014 to 0.050) | 0.281 |
| **Daily alcohol use Spline 1 with BMI** | -0.006 | 0.015 | -0.007 (-0.062 to 0.041) | 0.687 |
| **Daily alcohol use Spline 2 with BMI** | 0.012 | 0.011 | 0.019 (-0.014 to 0.051) | 0.258 |
| **Daily alcohol use Spline 1 with tobacco use** | -0.013 | 0.017 | -0.017 (-0.073 to 0.031) | 0.431 |
| **Daily alcohol use Spline 2 with tobacco use** | 0.013 | 0.010 | 0.024 (-0.013 to 0.060) | 0.203 |
| **Fit** | R^2^=0.05; AIC=24,932.92 | | | |

B=Unstandardized regression coefficient; SE B=Standard error for the unstandardized regression coefficient; β (95% CI)=Standardized regression coefficient and 95% Confidence Intervals.
